# Associations between commonly used patient-reported outcome tools in postpartum depression clinical practice and the Hamilton Rating Scale for Depression

**DOI:** 10.1007/s00737-020-01042-y

**Published:** 2020-07-14

**Authors:** Margaret E. Gerbasi, Adi Eldar-Lissai, Sarah Acaster, Moshe Fridman, Vijayveer Bonthapally, Paul Hodgkins, Stephen J. Kanes, Samantha Meltzer-Brody

**Affiliations:** 1 Sage Therapeutics, Inc., Cambridge, MA USA; 2 Acaster Lloyd Consulting Ltd., London, UK; 3 AMF Consulting, Los Angeles, CA USA; 4 Department of Psychiatry, University of North Carolina School of Medicine, 101 Manning Drive, Chapel Hill, NC 27514 USA

**Keywords:** Postpartum depression, Postpartum depression screening, Edinburgh Postnatal Depression Scale, Patient Health Questionnaire-9, Hamilton Depression Rating Scale

## Abstract

The objective of this study is to explore the associations between the patient-reported Edinburgh Postnatal Depression Scale (EPDS) and Patient Health Questionnaire (PHQ)-9 and clinician-reported 17-item Hamilton Depression Rating Scale (HAMD-17) in order to facilitate clinical decision-making. An integrated efficacy dataset of three randomized placebo-controlled trials (NCT02614547, NCT02942004, and NCT02942017) evaluating brexanolone injection, a neuroactive steroid chemically identical to allopregnanolone, in women with postpartum depression was used for this post hoc analysis. Data were pooled across treatment arms. Associations were assessed at day 30 (end-of-trial follow-up). Pearson correlation assessed the relationship between EPDS and PHQ-9 item and total scores and HAMD-17 total score. Cohen’s kappa assessed agreement of EPDS remission (score < 10) and PHQ-9 remission (score < 5) with HAMD-17 remission (score ≤ 7). Ordinary least squares (OLS) regression models were used to develop equations estimating HAMD-17 total scores from EPDS and PHQ-9 scores, respectively. The total scores showed large correlations (HAMD-17/EPDS: *r =* 0.71, *p <* 0.001; HAMD-17/PHQ-9: *r* = 0.75, *p <* 0.001). Individual EPDS and PHQ-9 items significantly correlated (*r*= 0.35 to 0.67, all *p <* 0.001) with HAMD-17 total score. EPDS had 79% sensitivity and 67% specificity to detect HAMD-17 remission; corresponding estimates for PHQ-9 were 76% and 78%. OLS models yielded the following equations: HAMD-17 total = 2.66 + (EPDS total × 0.87) and HAMD-17 total = 3.99 + (PHQ-9 total × 0.97). There were large and statistically significant associations between patient-reported outcomes (EPDS, PHQ-9) and clinician-reported outcomes (HAMD-17) as clinical improvements were associated with patient-reported symptom improvement. These results provide tools to help translate clinical trial data to clinical practice, thus aiding shared decision-making for this critical population.

## Introduction

Postpartum depression (PPD) is one of the most common medical complications during and after pregnancy, resulting in a substantial health-related quality of life burden for mothers, children, and partners (Callaghan et al. 2010; Centers for Disease Control and Prevention 2018; Centers for Disease Control and Prevention 2019; De Sisto et al. 2014; Ko et al. 2017; Martin et al. 2019; Moore Simas et al. 2019; Roberts et al. 2013; Vismara et al. 2016). In the USA, an estimated 11.5% of new mothers experience symptoms of PPD, with global estimates of 17.7% (Hahn-Holbrook et al. 2017; Ko et al. 2017).

Clinicians treating perinatal women commonly use the Edinburgh Postnatal Depression Scale (EPDS) or Patient Health Questionnaire (PHQ)-9 as PPD screening tools. Both the EPDS and PHQ-9 are patient-reported instruments and therefore provide unique perspective into the patient’s experience of their symptoms. Given the increasing focus on patient-centred care, understanding and incorporating patient’s voice into the assessment of health outcomes can facilitate shared decision-making, early diagnosis, individualized care, and patient satisfaction with treatment. The EPDS is a validated screening tool for PPD widely used to assess the likelihood of clinical depression and recently recommended by the American College of Obstetricians and Gynecologists (ACOG Committee Opinion No. 757 2018; Cox et al. 1987; Smith et al. 2016). Although the EPDS was developed as a screening tool, it is often used as an outcome measure in PPD studies and clinical practice (Appleby et al. 1997; McCabe-Beane et al. 2016; Sharp et al. 2010). The EPDS has been shown to perform well when compared to clinician-reported tools such as structured clinical interviews and more consistently than other patient-reported tools such as the Beck Depression Inventory (Beck et al. 1988; Bennett et al. 2004; Ji et al. 2011; Spinelli et al. 2013). The PHQ-9 is a general depression screening questionnaire that is also commonly used to screen for PPD, particularly in primary care settings, has been found to be highly specific for identifying PPD, and has comparable psychometrics, sensitivity, and specificity to the EPDS (Flynn et al. 2011; Gjerdingen et al. 2009; Kroenke et al. 2001; O'Connor et al. 2016; Sidebottom et al. 2012; Sit and Wisner 2009; Zhong et al. 2014).

The 17-item Hamilton Rating Scale for Depression (HAMD-17) is widely used as a primary endpoint in major depressive disorder (MDD) and PPD clinical trials and is commonly required by regulatory bodies (e.g. Clayton et al. 2015; Hantsoo et al. 2014; Papakostas et al. 2012). As an established clinician-reported outcome measure, it is regarded as the gold standard assessment in clinical trials, demonstrating stability over time and allowing for comparison of treatment effects between studies (Ji et al. 2011; Khan et al. 2002; Kim et al. 2014). However, given its complexity and length of time to complete, the HAMD-17 is less commonly used to assess depression in clinical practice. While EPDS and PHQ-9 severity group cut-off scores have been reported (Ji et al. 2011; Kroenke et al. 2001; McCabe-Beane et al. 2016), no mapping of EPDS or PHQ-9 scores to predict HAMD-17 scores has previously been reported. Understanding the direct relationship between patient-reported outcomes as measured either by the EPDS or by the PHQ-9 and the clinician-reported HAMD-17, based on evidence from a PPD-specific population, could further validate these screening tools in the identification of patients in need of treatment given symptom severity and enhance early diagnosis in this vulnerable population.

The present post hoc analysis aimed to bridge the existing interpretation gap and examine the relationship between the EPDS, PHQ-9, and HAMD-17 instruments. These post hoc analyses used an integrated dataset combining the data from three randomized, double-blind, trials of intravenous brexanolone compared to intravenous placebo in women with moderate and severe PPD (Kanes et al. 2017; Meltzer-Brody et al. 2018a). More specifically, using the HAMD-17 as the criterion measure, this study aimed to (1) assess the association between the current EPDS and PHQ-9 definitions of remission and the HAMD-17 definition of remission, (2) assess the association between the EPDS and PHQ-9 item and total scores with the HAMD-17 total score, (3) develop a mapping equation to permit the estimation of HAMD-17 scores from EPDS and PHQ-9 scores.

## Methods

### Study design and participants

The current report utilizes data from phase 2 and phase 3 clinical trials, which examined the safety and efficacy of intravenous brexanolone compared with intravenous placebo in patients with moderate and severe PPD (clinicaltrials.gov identifiers NCT02614547, NCT02942004, and NCT02942017). Full descriptions of the trial designs and inclusion and exclusion criteria have been published previously (Kanes et al. 2017; Meltzer-Brody et al. 2018a). Briefly, one phase 2 (study A) and two phase 3 (studies B and C) multicentre, randomized, double-blind, placebo-controlled trials were conducted across the USA under an umbrella protocol. All trials enrolled female participants; aged 18–45 years; in good physical health; had a major depressive episode with onset no earlier than the third trimester and no later than 4 weeks after delivery, as determined by the Structured Clinical Interview for Diagnostic and Statistical Manual of Mental Disorders-IV Axis I Disorders (First et al. 1996); had a HAMD-17 total score ≥ 26 (severe PPD, studies A and B) or 20–25 (moderate PPD, study C); and were 6 months postpartum or less at screening. Patients were either randomized 1:1:1 to receive 60-h infusion of brexanolone 90 μg/kg/h, brexanolone 60 μg/kg/h, or placebo (study B), or 1:1 to receive 90 μg/kg/h brexanolone or placebo (studies A and C). Patients who received the recommended target dose for brexanolone, 90 μg/kg/h, or placebo were included in the integrated efficacy dataset, employed for these analyses.

Patients were treated in a medically supervised setting for 72 h: 60 h of study drug infusion and 12 h for completion of assessments. Patients were followed until day 30, with clinical and safety assessments at days 7 and 30. The EPDS, PHQ-9, and HAMD-17 were administered at baseline, hour 60, day 7, and day 30 for all patients. The primary endpoint in each trial was the least-squares mean difference in change from baseline in HAMD-17 total score at hour 60.

### Measures

The present analysis included three clinical outcome assessments: the patient-reported EPDS, patient-reported PHQ-9, and clinician-reported HAMD-17. The psychometric properties of these scales have been extensively described in the literature (Bagby et al. 2004; Flynn et al. 2011; Hamilton 1960; Kroenke et al. 2001; McCabe-Beane et al. 2016; Zhong et al. 2014).

The EPDS is a 10-item patient-reported outcome measure of depressive symptom severity specific to the perinatal period, originally developed as a screening tool. The EPDS has a recall period of 7 days. Items are scored on a 0–3 scale and summed to compute a total score, which ranges from 0 to 30, with higher scores indicating more severe depression. A score of 10 has been recommended as a cut-off point for the presence of minor PPD, and score of 13 and above for major PPD in English-speaking women (Cox et al. 1987; Harris et al. 1989; Murray and Carothers 1990). Remission was defined as an EPDS score of < 10 in the present analysis.

The PHQ-9 is a 9-item patient-reported outcome and depression screening tool. The PHQ-9 has a recall period of 14 days. Items are scored on a 0 (not at all) to 3 (nearly every day) scale and summed for a total score ranging from 0 to 27. Remission or minimal symptom severity has been defined as a score of < 5 (Kroenke et al. 2001).

The HAMD-17 is clinician-reported scale that evaluates core symptoms of depression. Items are scored on a 0 (none/absent) to 4 (most severe) or 0 (none/absent) to 2 (severe) scale (Hamilton 1960). Individual item scores are summed to compute the total score, which ranges from 0 to 52, with higher scores indicating more severe depression. Clinical remission is defined as a HAMD-17 total score of ≤ 7.

### Statistical analysis

The analysis was conducted on the integrated efficacy dataset. The efficacy dataset included all randomized patients who started the infusion of study drug and who had a valid baseline HAMD-17 assessment and at least one post baseline HAMD-17 assessment. As the objective of this analysis was to examine the relationship between the EPDS, PHQ-9, and HAMD-17, data was pooled across treatment arms. Furthermore, only the day 30 assessment point data (end of study) was utilized. Due to study inclusion criteria, the data at baseline and earlier assessment points showed less variability, and variability is required when trying to establish a relationship between measures (Salkind 2010).

Patients with day 30 EPDS, PHQ-9, and HAMD-17 data were included in the analytical sample for the present analysis. The sample characteristics were summarized descriptively.

To examine the association EPDS remission (score < 10) and PHQ-9 remission (score < 5) with HAMD-17 remission (score ≤ 7), the proportions of EPDS and PHQ-9 remitters were compared with the proportion of HAMD-17 remitters using Fisher’s exact test; concordance was tested using the Kappa coefficient. Kappa were interpreted as poor (< 0), slight (0–0.2), fair (> 0.2–0.4), moderate (> 0.4–0.6), or substantial (> 0.6–0.8) (Landis and Koch 1977).

Pearson correlations were calculated to examine the association of EPDS and PHQ-9 total scores with HAMD-17 total score. As a validity check, as well as to inform multivariable modelling, the correlations between the EPDS and PHQ-9 items and the HAMD-17 total score were assessed for inconsistent or disproportionate values. The correlations were interpreted as small (0.3–< 0.5), moderate (0.5–< 0.7), or large (≥ 0.7) (Hinkle et al. 2003).

Ordinary least squares regression models were used to develop equations to estimate HAMD-17 total score from EPDS and PHQ-9 total score, respectively. For each screening tool, three models were applied: (1) a simple regression of the HAMD-17 and EPDS/PHQ-9 total score (model 1), (2) model 1 with a quadratic term added (EPDS/PHQ-9 total score squared; model 2), (3) a stepwise fivefold cross-validation predicted residual error sum of squares (CV PRESS) model using EPDS/PHQ-9 items to predict HAMD-17 total score (model 3). For model 3 using cross-validation methods as a means of preventing overfitting, cross-validated covariates were selected from the 10 EPDS items and 9 PHQ-9 items, using CV PRESS as the stepwise selection and stop criteria. Age and a combined PPD history/nulliparity variable (first pregnancy; pregnancy history with PPD, pregnancy history without PPD) were initially included as covariates but were removed as found non-significant to keep the models as simple as possible for potential use in a clinical practice setting.

Two-sided tests were used and *p* values of ≤ 0.05 were considered statistically significant. Statistical significance was not adjusted for multiple comparisons. SAS version 9.4 (SAS Institute Inc., Cary, NC, USA) was used for all statistical analysis.

## Results

In total, 199 patients provided EPDS, PHQ-9, and HAMD-17 data at day 30 and were thus eligible for the present analysis. While all analyses were conducted on the pooled sample, the patient demographics and baseline characteristics are presented overall and by treatment arm for information (Table 1). Of the 199 patients included in these analyses at baseline, most patients were white (121, 61%), 74 (37%) were black or African-American, and 35 (18%) were Hispanic or Latino. A higher proportion of patients had onset of PPD within 4 weeks of delivery than the third trimester. The mean EPDS total score at day 30 was 9.04 (SD = 6.84), the mean PHQ-9 total score was 6.76 (SD = 6.52), and the mean HAMD-17 total score was 10.56 (SD = 8.47), with minimum and maximum scores 0–27, 0–25, and 0–31, respectively.

**Table 1:** Baseline demographics and characteristics

Characteristics	Placebo (*n* = 105)	Brexanolone (*n* = 94)	Overall (*n* = 199)
Age, years, mean (SD)	27.45 (5.90)	27.95 (5.98)	27.68 (5.93)
Ethnicity, *n* (%)			
Hispanic or Latino	19 (18%)	16 (17%)	35 (18%)
Not Hispanic or Latino	86 (82%)	78 (83%)	164 (82%)
Race, *n* (%)			
Black or African-American	40 (38%)	34 (36%)	74 (37%)
White	63 (60%)	58 (62%)	121 (61%)
Other	2 (2%)	2 (2%)	4 (2%)
Height, cm, Mean (SD)	163.62 (8.23)	164.15 (6.57)	163.87 (7.48)
Weight, kg, Mean (SD)	83.89 (23.88)	85.66 (23.90)	84.72 (23.84)
Body-mass index, kg/m^2^, Mean (SD)	31.24 (8.19)	31.68 (8.23)	31.45 (8.19)
Personal history of psychiatric disorders, *n* (%)			
Depression	42 (40%)	30 (32%)	72 (36%)
Anxiety	34 (32%)	34 (36%)	68 (34%)
Premenstrual dysphoric disorder	3 (3%)	6 (6%)	9 (5%)
Substance abuse	0	1 (1%)	1 (0%)
Schizophrenia	5 (5%)	2 (2%)	7 (4%)
Previous PPD	35 (33%)	26 (28%)	61 (31%)
Family history of PPD, *n* (%)	23 (22%)	31 (33%)	54 (27%)
Onset of PPD, *n* (%)			
Third trimester	29 (28%)	19 (20%)	48 (24%)
Within 4 weeks of delivery	76 (72%)	75 (80%)	151 (76%)
Antidepressant use at baseline, *n* (%)	25 (24%)	19 (20%)	44 (22%)
Other measures at baseline^a^, mean (SD, *n*)			
HAMD-17 total	25.70 (3.66, *n* = 105)	25.40 (3.38, *n* = 94)	25.56 (3.53, *n =* 199)
EPDS total	19.88 (3.94, *n* = 103)	19.58 (3.95, *n* = 92)	19.74 (3.93, *n* = 195)
PHQ-9 total	17.75 (5.06, *n* = 102)	17.87 (4.63, *n* = 91)	17.80 (4.85, *n* = 193)

Weight, height and body mass index data were measured at screening

*PPD* post-partum depression, *HAMD-17* 17-item Hamilton Rating Scale for Depression, *EPDS* Edinburgh Post Natal Depression Scale, *PHQ-9* Patient Health Questionnaire-9

^a^Assessed before infusion on day 1. Ten patients without complete day 30 data had similar baseline HAMD-17, EPDS, and PHQ-9 total scores to the 199 patients included in analytic sample

### Association EPDS and PHQ-9 remission with HAMD-17 remission

There were statistically significant associations between EPDS and PHQ-9 definitions of remission with HAMD-17 remission (Table 2), both with moderate Kappa agreement. Among patients classified as remitters according to the HAMD-17 definition, 79% and 76% were also classified as remitters by the EPDS and PHQ-9 definitions, respectively (sensitivity). Among patients classified as a non-remitter on the HAMD-17, 67% and 78% were also classified as such by the EPDS and PHQ-9, respectively (specificity).

**Table 2:** Associations between HAMD-17 remitters and EPDS and PHQ-9 remitters at day 30

	HAMD-17 remission		Fisher’s exact test	Kappa (95% CI)
	Yes	No		
EPDS remission				
Yes	72 (79%)	36 (33%)	*p* < 0.001	0.45 (0.33, 0.57)
No	19 (21%)	72 (67%)		
PHQ-9 remission				
Yes	69 (76%)	24 (22%)	*p* < 0.001	0.54 (0.42, 0.65)
No	22 (24%)	84 (78%)		

*HAMD-17* 17-item Hamilton Rating Scale for Depression, remission ≤ 7, *EPDS* Edinburgh Post Natal Depression Scale, remission < 10, *PHQ-9* Patient Health Questionnaire-9, remission <5, *CI* confidence interval

### Association of EPDS and PHQ-9 total and item scores with HAMD-17 total score

All correlations of EPDS and PHQ-9 total and items scores with HAMD-17 total score were statistically significant (*p*s < 0.001; Table 3). The total scores showed large correlations (EPDS/HAMD-17*: r =* 0.71; PHQ-9/HAMD-17: *r* = 0.75). Item scores were moderately correlated with HAMD-17 total score except ‘thought of self-harm’ for both EPDS and PHQ-9 and ‘able to laugh’ and ‘looked forward with enjoyment’ for EPDS*.*

**Table 3:** Pearson correlations between the HAMD-17 Total and EPDS and PHQ-9 total and items at day 30 (all *p*’s < 0.001)

	Correlation (*r*) with HAMD-17 total (95% CI) (*n* = 199)
EPDS	
Total score	0.71 (0.63, 0.77)
Able to laugh	0.42 (0.30, 0.53)
Looked forward with enjoyment	0.44 (0.32, 0.54)
Blamed myself unnecessarily	0.58 (0.47, 0.66)
Anxious or worried for no good reason	0.51 (0.40, 0.61)
Scared or panicky no very good reason	0.50 (0.39, 0.60)
Things getting to me	0.61 (0.51, 0.69)
So unhappy had difficulty sleeping	0.55 (0.44, 0.64)
Sad or miserable	0.67 (0.59, 0.74)
So unhappy that I have been crying	0.54 (0.44, 0.63)
Thoughts of self-harm	0.35 (0.22, 0.47)
PHQ-9	
Total score	0.75 (0.68, 0.80)
Little interest or pleasure in doing things	0.65 (0.56, 0.72)
Feeling down, depressed, or hopeless	0.65 (0.57, 0.73)
Trouble falling or staying asleep, or sleeping too much	0.57 (0.47, 0.66)
Feeling tired or having little energy	0.62 (0.53, 0.70)
Poor appetite or overeating	0.58 (0.48, 0.67)
Feeling bad about yourself	0.62 (0.53, 0.70)
Trouble concentrating	0.58 (0.48, 0.67)
Moving or speaking slowly or being fidgety or restless	0.43 (0.31, 0.53)
Thoughts of self-harm	0.35 (0.22, 0.46)

*HAMD-17* 17-item Hamilton Rating Scale for Depression, *EPDS* Edinburgh Post Natal Depression Scale, *PHQ-9* Patient Health Questionnaire-9, *CI* confidence interval

### Estimating HAMD-17 total score from EPDS and PHQ-9

As shown in Table 4, the additional complexity of the quadratic term (model 2) and CV PRESS item-level model (model 3) did little to improve the variance explained statistics or reduce the prediction error, making model 1 the most parsimonious and recommended model to estimate HAMD-17 total score for both the EPDS and PHQ-9. Age and PPD/nulliparity were not significant predictors in any models (*p* > 0.05) and were thus removed. Based on model 1, the following equation can be used to estimate HAMD-17 total score from EPDS total score: HAMD-17 total = 2.66 + (EPDS total × 0.87). HAMD-17 total score can be estimated from PHQ-9 total score with a similar equation: HAMD-17 total = 3.99 + (PHQ-9 total × 0.97). This results in an integer range of estimated HAMD-17 total scores of 3–29 from the EPDS [2.66 + (0 / 30 × 0.87)] and 4–30 from the PHQ-9 [3.99 + (0 / 27 × 0.97)], very much aligned with the observed range of HAMD-17 total score (0–31)*.*

**Table 4:** Regression models to predict HAMD-17 total scores from EPDS and PHQ-9 scores

	Model 1: simple total score OLS		Model 2: model 1 + quadratic term		Model 3: CV PRESS with items	
	Coefficient	95% CI	Coefficient	95% CI	Coefficient	95% CI
EPDS						
Intercept	2.66***	1.27, 4.05	3.26***	1.54, 4.97	2.66***	1.28, 4.05
Total score	0.87***	0.75, 1.00	0.66***	0.28, 1.04	–	–
Total score quadratic	–	–	0.01	− 0.01, 0.03	–	–
Item—able to laugh	–	–	–	–	0.94	− 0.27, 2.16
Item—blamed myself unnecessarily	–	–	–	–	1.34*	0.24, 2.44
Item—things getting to me	–	–	–	–	1.65**	0.42, 2.88
Item—sad or miserable	–	–	–	–	3.19***	1.97, 4.41
Item—thoughts of self-harm	–	–	–	–	1.70*	0.02, 3.37
*R*^2^	0.50		0.50		0.53	
Adjusted *R*^2^	0.50		0.50		0.52	
RMSE	6.01		6.01		5.86	
PHQ-9						
Intercept	3.99***	2.85, 5.12	3.00***	1.61, 4.40	4.16***	3.01, 5.31
Total score	0.97***	0.85, 1.09	1.40***	1.02, 1.78	–	–
Total score quadratic	–	–	− 0.02*	− 0.04, − 0.003	–	–
Item—little interest or pleasure in doing things	–	–	–	–	1.46*	0.04, 2.88
Item—poor appetite or over eating	–	–	–	–	1.65*	0.36, 2.95
Item—feeling down, depressed, or hopeless	–	–	–	–	1.31**	0.36, 2.26
Item—trouble concentrating	–	–	–	–	1.91***	0.94, 2.89
Item—trouble falling or staying asleep, or sleeping too much	–	–	–	–	1.48*	0.27, 2.69
*R*^2^	0.56		0.57		0.56	
Adjusted *R*^2^	0.56		0.57		0.55	
RMSE	5.64		5.57		5.68	

Model 1 includes EPDS/PHQ-9 total score only, Model 2 includes EPDS/PHQ-9 total score and squared total score, Model 3 uses CV PRESS selection and stop criteria to fit model based on EPDS/PHQ-9 individual items

*HAMD-17* 17-item Hamilton Rating Scale for Depression, *EPDS* Edinburgh Post Natal Depression Scale, *PHQ-9* Patient Health Questionnaire-9, *OLS* ordinary least squares, *CI* confidence interval, *CV PRESS* cross-validation predicted residual error sum of squares, *RMSE* root mean squared error

*p value < 0.05; **p value < 0.01; ***p value < 0.001

Italicized coefficients correspond to the selected models

## Discussion

PPD is frequently unrecognized, undiagnosed, and inadequately treated (Cox et al. 2016). A more nuanced understanding of how commonly used screening tools such as the EPDS and PHQ-9 relate to clinical trial outcomes, such as the HAMD-17, may help address failures in diagnosis and treatment. The observed large associations between EPDS and PHQ-9 total score and HAMD-17 total score, as well as moderate associations of remission definitions, suggest that patients’ self-reports of their symptoms generally align with clinician-ratings, such as the HAMD-17, that may be prohibitively time and training intensive to obtain and further validate the ability of these screening tools to identify patients with PPD symptoms. Once a patient is diagnosed, management generally follows a stepped care approach, wherein patients with mild symptoms are treated through low-intensity interventions and those with symptoms that do not respond, are more severe, or present acutely are typically offered pharmacologic interventions, either alone or adjunctive to low-intensity interventions (Meltzer-Brody et al. 2018b). The observed pattern of results in these analyses, wherein higher scores on EPDS and PHQ-9 are associated with more severe symptoms as assessed by HAMD-17, may help clinicians who have identified and diagnosed PPD patients to understand and discuss with the patients what treatments are likely appropriate. Primary care physicians, obstetricians, and gynaecologists and even paediatricians who administer an EPDS or PHQ-9 may have a better ability to recognize which women are likely to receive benefit from low-intensity interventions, such as psychosocial support, and those that may warrant prompt referral for more intensive care. Clinicians seeking to practice shared decision-making regularly need to draw on and interpret a high volume of new clinical evidence in order to describe and discuss available options, alongside possible benefits or harms (Price 2005). Being able to understand and translate the relationship between the HAMD-17 data commonly reported as evidence of treatment efficacy in clinical trials and the EPDS or PHQ-9 data being captured in clinic will hopefully assist clinicians in more confidently and readily interpreting trial evidence to present during shared decision discussions. Given limitations in healthcare resources and availability of specialists, confidence in the use of these tools for valid screening and treatment decision-making may also help ameliorate stresses to the healthcare system.

The large associations observed between total scores are notable given that the measures differ in terms of patient versus clinician-rating, as well as in content, with EPDS designed specifically for the perinatal period, PHQ-9 initially designed for general depression, and HAMD-17 designed to assess changes in symptom severity. The moderate associations observed between EPDS and PHQ-9 item scores and HAMD-17 total score are in line with expectations, as individual item scores are expected to show more variability than summed total scores. Furthermore, as an advance to the current reliance upon individual outcome measure cut-off values to interpret scores, the current study also conducted regression analysis to provide two simple mapping equations that can easily be applied in any clinical setting.

### Limitations

While the present analysis advances current understanding and interpretation of EPDS, PHQ-9, and HAMD-17, these analyses are based on a clinical trial population, and the generalizability to the broader population should be examined through validation of this work in an external dataset. For example, it may be of interest to validate the mapping equations on a sample including a broader range of HAMD-17 total score. While the current sample included EPDS and PHQ-9 total score from almost the full range, 0–25/27 and 0–27/30, respectively, the HAMD-17 total score range was 0–31/52. However, as any HAMD-17 total score higher than 25 indicates severe depression, the full range of depression severity required for clinical decision-making was covered in the present analysis. Indeed, the maximum baseline HAMD-17 total score in the severe PPD patients included in this analysis was 38, as has been reported in severe PPD elsewhere (Nonacs et al. 2005), suggesting that the higher values may be less common in PPD. The baseline data was not included in the analysis reported here due to the restriction in range of PHQ-9, EPDS, and HAMD-17 total score as a result of the trial inclusion criteria for moderate/severe PPD.

## Conclusions

The present post hoc analysis demonstrates large and statistically significant associations between patient-reported EPDS and PHQ-9 scores and the clinician-reported HAMD-17 and in an integrated efficacy dataset of PPD trials and provides the first mapping equations to permit the estimation of HAMD-17 total score from known EPDS or PHQ-9 scores. Given the role of the HAMD-17 as the gold standard criterion for evaluating the efficacy of treatments in clinical trials, the results presented here provide tools to aid interpretation of clinical trial data in clinical practice, thus aiding informed decision-making for multiple stakeholders in regulatory and clinical settings and shared decision-making in clinical practice for this critical population of women with PPD.
